# Resveratrol improves glycemic control in insulin-treated diabetic rats: participation of the hepatic territory

**DOI:** 10.1186/s12986-016-0103-0

**Published:** 2016-06-29

**Authors:** Caio Yogi Yonamine, Erika Pinheiro-Machado, Maria Luiza Michalani, Helayne Soares Freitas, Maristela Mitiko Okamoto, Maria Lucia Corrêa-Giannella, Ubiratan Fabres Machado

**Affiliations:** Department of Physiology and Biophysics, Institute of Biomedical Sciences, University of São Paulo, Av. Prof. Lineu Prestes, 1524, 05508-000 São Paulo, SP Brazil; Laboratory of Medical Investigation 18 (LIM-18) and Cell and Molecular Therapy Center (NUCEL), Medical School, University of São Paulo, São Paulo, Brazil

**Keywords:** Type 1 diabetes, Glucose transporters, Glycogen, Gluconeogenic enzymes, Liver metabolism, SIRT1, GLUT2

## Abstract

**Background:**

Resveratrol is a natural polyphenol that has been proposed to improve glycemic control in diabetes, by mechanisms that involve improvement in insulin secretion and activity. In type 1 diabetes (T1D), in which insulin therapy is obligatory, resveratrol treatment has never been investigated. The present study aimed to evaluate resveratrol as an adjunctive agent to insulin therapy in a T1D-like experimental model.

**Methods:**

Rats were rendered diabetic by streptozotocin (STZ) treatment. Twenty days later, four groups of animals were studied: non-diabetic (ND); diabetic treated with placebo (DP); diabetic treated with insulin (DI) and diabetic treated with insulin plus resveratrol (DIR). After 30 days of treatment, 24-hour urine was collected; then, blood, soleus muscle, proximal small intestine, renal cortex and liver were sampled. Specific glucose transporter proteins were analyzed (Western blotting) in each territory of interest. Solute carrier family 2 member 2 (*Slc2a2*), phosphoenolpyruvate carboxykinase (*Pck1*) and glucose-6-phosphatase catalytic subunit (*G6pc*) mRNAs (qPCR), glycogen storage and sirtuin 1 (SIRT1) activity were analyzed in liver.

**Results:**

Diabetes induction increased blood glucose, plasma fructosamine concentrations, and glycosuria. Insulin therapy partially recovered the glycemic control; however, resveratrol as adjunctive therapy additionally improved glycemic control and restored plasma fructosamine concentration to values of non-diabetic rats. Resveratrol did not alter the expression of the glucose transporters GLUT2 and SGLT1 in the intestine, GLUT2 and SGLT2 in kidney and GLUT4 in soleus, suggesting that fluxes of glucose in these territories were unaltered. Differently, in liver, resveratrol promoted a reduction in *Slc2a2*, *Pck1*, and *G6pc* mRNAs, as well as in GLUT2 protein (*P* < 0.05, DIR vs. DI); besides, it increased (*P* < 0.01, DIR vs. DI) the hepatic glycogen content, and SIRT1 protein.

**Conclusions:**

Resveratrol is able to improve glycemic control in insulin-treated T1D-like rats. This effect seems not to involve changes in glucose fluxes in the small intestine, renal proximal tubule, and soleus skeletal muscle; but to be related to several changes in the liver, where downregulation of *Slc2a2*/GLUT2, *Pck1*, and *G6pc* expression was observed, favoring reduction of glucose production and efflux. Besides, resveratrol increased SIRT1 nuclear protein content in liver, which may be related to the observed gene expression regulations.

## Background

Diabetes mellitus (DM) is an epidemic metabolic disease growing at exponential rate. Type 2 DM (T2D) accounts for around 90 % of all diabetes subjects, but type 1 DM (T1D) incidence is also increasing worldwide [1]. The failure of pancreatic beta cells to produce insulin, and the impairment of insulin action, play a central role in the disruption of glycemic homeostasis, leading to hyperglycemia, a hallmark of DM [2]. DM shows a complex scenario, including complications derived from the macro and microangiopathy development [3]. At present, optimized glucose control is recognized as the best approach to reduce the risk of diabetes chronic complications [4].

Insulin sensitizer agents, which could improve glycemic control, have been extensively investigated for treatment of T2D. However, the recent finding of insulin resistance in T1D patients [5, 6] has driven attention to their use as adjunctive agents to insulin therapy [7–10]. Beyond several compounds tested to treat DM, resveratrol gained much attention in recent years [11]. Resveratrol is a natural polyphenol that belongs to stilbene class, widely found in several plant species, especially in grapes and blueberries [12]. It is capable of activating NAD^+^ -dependent histone deacetylase sirtuin 1 (SIRT1), the main mechanism related to its effects [11]. Numerous studies report a wide diversity of healthy-related properties of resveratrol, including management of diseases such as cancer, Alzheimer, obesity and DM [13].

Regarding the potential effects of resveratrol in DM treatment, a glycemia-lowering effect was described in normal rats and mice [14, 15], high-fat fed mice [16], T2D *db/db* mice [17], and T2D humans as well [11]. In these conditions, a reduction in insulin resistance has been described, and in some of the experimental models, a concomitant increase in insulin secretion was also observed [15, 17]. However, it remains unknown whether improved glycemic control was cause or consequence of improved beta-cell function. Curiously, there are some reports suggesting that resveratrol could also decrease hyperglycemia in streptozotocin (STZ)-induced diabetic rats [14, 18–20], an insulinopenic model of DM considered a T1D-like condition, in which amelioration of pancreatic insulin secretion would be unexpected.

Glycemic homeostasis results from an orchestrated regulation of territorial glucose fluxes, which includes flows into and out of the extracellular/blood compartments [21, 22]. Some of these fluxes of glucose are highly variable, even being tightly regulated, and they can alter blood glucose quite rapidly. These include glucose fluxes to blood from the intestine (postprandial absorption), liver (glucose production) and kidney (glucose reabsorption); and also glucose fluxes from blood to liver, skeletal muscle and adipose tissue, highlighting these fluxes as the most variable and regulatable [21–23]. All these fluxes involve several distinct and complex mechanisms, and, in each territory, one or more glucose transporter isoforms play a key role [24, 25]. In epithelial cells of proximal intestine and in renal proximal tubule, sodium glucose cotransporter 1 and 2 (SGLT1 and SGLT2), respectively, uptake glucose at the luminal membrane; whereas the facilitative glucose transporter 2 (GLUT2) effluxes glucose into the interstitium/blood side [24, 25]. In hepatocytes, GLUT2 performs a bidirectional flux of glucose, accordingly to the substrate concentration gradient, which is critical for cellular glucose production [23]. Finally, the glucose uptake by muscle and adipose tissue occurs through the GLUT4, which can be acutely translocated to the plasma membrane in response to insulin [24, 26].

Most of these glucose fluxes have been proposed to be altered in DM, and that would involve changes in the expression of specific glucose transporters. On the other hand, regulation of some glucose transporters has been proposed as important targets for the development of preventive and therapeutic approaches for DM [23, 26, 27]. In this context, resveratrol could modulate the expression of some GLUTs/SGLTs spread in many peripheral territories, and that might participate in its effect on glycemic homeostasis.

So far, the beneficial effects of resveratrol have been shown in rodent models of T2D, in T2D patients, and in untreated T1D-like rats. This latter condition does not contribute to the investigation of the potential benefits of resveratrol for T1D patients, because it does not reflect their real life situation, since they necessarily require insulin therapy. Thus, the present study aimed to investigate if resveratrol could act as an adjunctive agent to insulin therapy in a T1D-like experimental model. For that, insulin-treated STZ-rats were additionally treated with resveratrol; glycemic control and expression of glucose transporters in distinct territories involved on glycemic homeostasis were evaluated. Besides, because the hepatic GLUT2 expression was highly altered, glucose metabolism markers involved in the regulation of glucose fluxes and SIRT1 activity were also investigated in this territory.

## Methods

### Animals and treatments

Forty 60-day old male Wistar rats weighing 250 g were obtained from the Animal Center of the Institute of Biomedical Sciences, University of São Paulo. The animals were housed in a room kept at constant temperature (23 ± 2 °C), in light/dark cycle (12/12 h), receiving standard rat chow (Nuvilab CR1; Nuvital Nutrients S/A, Colombo, Paraná, Brazil) and tap water *ad libitum*.

At 75 days of life, animals were rendered diabetic by intravenous injection of STZ (Sigma Chemical Co, St Louis, MO, EUA) at a dose of 50 mg/Kg of body weight, solubilized in citrate buffer (pH 4.5); and control rats were injected with citrate buffer. The procedure was performed in halothane (Tanohalo®, Cristália, Itapira, SP, Brazil) anaesthetized animals.

Twenty days later (95-day old rats), a trial was performed to evaluate the efficiency of diabetes induction, and animals with a blood glucose concentration above 300 mg/dL (measured at 10:00 AM, after 4-hour food deprivation) were included in the study. Immediately, the animals were separated into four groups: non-diabetic control (ND); diabetic treated with 0.9 % NaCl as placebo (DP); diabetic treated with 5 U/day NPH insulin (Humulin®, Eli Lilly and Company, Indianapolis, IN, USA) (DI) and diabetic treated with 5 U/day NPH insulin plus 10 mg/Kg body weight resveratrol (Sigma-Aldrich, St. Louis, USA) (DIR). Insulin and resveratrol were subcutaneously and intraperitoneally injected, respectively. The treatments were conducted during thirty days, totalizing 50 days of diabetes duration. The experimental protocol was approved by the Ethical Committee for Animal Research of the Institute of Biomedical Sciences, University of São Paulo (#194/2013).

### Sampling collection

At the end of the treatments, 24-hour urine was collected. Then, at 10:00 AM (after 4-hour food deprivation), the animals were anaesthetized with 60 mg/Kg sodium thiopental (Cristália®, Itapira, São Paulo, Brazil), and tail blood samples were collected for glucose concentration analysis. After, soleus muscle, liver, kidney and proximal small intestine were sampled and storage at –70 °C for further analysis. Additionally, immediately after liver sampling, blood was collected from the inferior vena cava, for fructosamine concentration analyzes in plasma. Kidneys were removed, and outside cortex slices were excised, as previously described [28].

### Plasma fructosamine, blood glucose, and 24-hour urinary glucose excretion

Plasma fructosamine concentration was measured by a kinetic-colorimetric assay (Frutosamina, Labtest, Lagoa Santa, MG, Brazil) and blood glucose concentration by a glucometer (Accu-Check Active Basel, Switzerland). The 24-hour urine volume was measured, a sample was centrifuged at 1,000 g (10 min), and the supernatant was used to measure glucose concentrations by an enzymatic-colorimetric assay (Glicose Liquiform Labtest, Lagoa Santa, MG, Brazil); the results were expressed as 24-hour glucose excretion, taking into account the total urinary volume.

### Hepatic glycogen content

Hepatic glycogen content was measured as previously described [29]. Briefly, 250 mg of liver tissue were treated for glycogen breakdown, and thus, glycogen was pelleted by ethanol/Na_2_SO_4_ treatment. After hydrolysis, glycogen content was calculated based on the glucose concentrations in the samples, measured by an enzymatic-colorimetric assay (Glicose Liquiform, Labtest, Lagoa Santa, MG, Brazil).

### mRNA quantification by real-time polymerase chain reaction (qPCR)

Total RNA was isolated from approximately 100 mg of soleus skeletal muscle, and liver. The samples were processed accordingly to TRIzol® Reagent manufacturer specifications (Invitrogen, Carlsbad, CA, USA). The amount of total RNA in each sample was determined using a spectrophotometer (Gene Quant, PHARMACIA BIOTECH - Biochrom, Cambridge, UK). The integrity of RNA was verified by the presence of 18S and 28S bands and confirmed by a 1.5 % denaturant agarose gel electrophoresis exposed to ultra-violet light (Epi Chemi II Darkroom, UVP BioImaging Systems, Upland, California, CA, USA). The reverse transcriptase (RT) reaction was performed from 2 μg of total RNA, adding to the reaction: oligo dT (100 μg/mL), 10 mM of each dNTP, 5X First-Strand buffer and 2 μl (200 U/μl) of M-MLV Reverse Transcriptase (Promega, Madison, WI). The conditions of RT reaction were 65 °C for 10 min, followed by 37 °C for 60 min, and 95 °C for 10 min. The qPCR amplification was performed using Taqman® PCR master mix kit (Applied Biosystems Inc., Foster City, CA, USA) and carried out with StepOne Plus Instrument (Applied Biosystems Inc., Foster City, CA, USA). The PCR conditions were 1 cycle of 10 min at 95 °C and 40 cycles of 30 s at 95 °C, 60 s at 60 °C and 60 s at 72 °C. The method of 2 ^–ΔΔCt^ was adopted for analysis. The genes analyzed were: solute carrier family 2 member 4 (*Slc2a4*), solute carrier family 2 member 2 (*Slc2a2*), phosphoenolpyruvate carboxykinase 1 (*Pck1*), glucose-6-phosphatase catalytic subunit (*G6pc*). The reference gene used was beta-2-microglobulin (*B2m*), accordingly to RefFinder software analysis. The used primers are depicted in Table 1.

**Table 1:** Details for the primers and identification (ID) codes of the Taqman Gene Expression Assays used for real-time polymerase chain reaction (qPCR)

Gene	Primers sequence	Dye	Assay ID
*Slc2a4*	Sense: 5’-GGC TGT GCC ATC TTG ATG AC-3’	FAM	AI5IQJM 186914021_1
	Anti-sense: 5’-CAC GAT GGA CAC ATA ACT CAT GGA T-3’		
*Slc2a2*	Inventoried	FAM	Rn00563565_m1
*Pck1*	Inventoried	FAM	Rn01529014_m1
*G6pc*	Inventoried	FAM	Rn00689876_m1
*B2m*	Inventoried	FAM	Rn00560865_m1

*Slc2a4* solute carrier family 2 member 4, *Slc2a2* solute carrier family 2 member 2, *Pck1* phosphoenolpyruvate carboxykinase 1, *G6pc* glucose-6-phosphatase catalytic subunit, *B2m* beta-2-microglobulin

### Evaluation of protein expression by Western blotting

Membrane fractions were prepared as previously described [28, 30, 31]. Liver and kidney samples were processed in the same way. The samples were homogenized in buffer solution (100 mM Tris pH 7.5, 10 mM EDTA, 10 % SDS, 10 mM sodium fluoride, 10 mM sodium pyrophosphate and 10 mM sodium orthovanadate); the homogenate was centrifuged at 1,200 g (15 min), the resulting supernatant was centrifuged at 12,000 g (20 min), and the final pellet was suspended in the same buffer as an enriched plasma membrane fraction. Soleus muscle samples were homogenized in the same buffer, centrifuged at 760 g (10 min), and the supernatant was directly used as a total membrane fraction (plasma membrane and microsomes). Proximal small intestine samples were homogenized in the same buffer, centrifuged at 3,000 g (10 min), the supernatant was centrifuged at 12,000 g (30 min); and the pellet was used as an enriched plasma membrane fraction.

Total protein content in the samples was determined by Bradford method (Bio-Rad Laboratories, Hercules, CA, USA). Equal amounts of protein (40 to 60 μg, accordingly to the tissue) were electrophoresed, transferred to nitrocellulose membrane and immunoblotted, accordingly to the tissue, with anti-GLUT4 (EMD Millipore, Billerica, MA, USA, #07-1404), anti-GLUT2 (EMD Millipore, #07-1402), anti-SGLT1 (Millipore, #07-1417) and anti-SGLT2 (Santa Cruz, Dallas, TX, USA, #98975) antibodies. The appropriate secondary conjugated antibody was used accordingly to manufacturer specifications, followed by enhanced chemiluminescence (ECL) procedure. The optical density of the blots was analyzed using Image J software (National Institutes of Health, Bethesda, MD, USA), and the densities of the respective lanes, stained by Ponceau, were used for normalization. The results were expressed as arbitrary units, related to mean of the controls, which was set as 1.0.

### Nuclear content of SIRT1

Nuclear proteins were extracted from liver samples as previously described [5, 32]. Briefly, 0.3 g of liver tissue was pulverized in nitrogen, and suspended in ice-cold phosphate buffer saline with 0.2 mM dithiothreitol and 0.2 mM phenylmethylsulphonyl fluoride. After 1,000 g centrifugation (10 min), the pellet was incubated in a hypotonic buffer (10 min), followed by 12,000 g centrifugation (1 min). The pellet was resuspended in a high salt concentration buffer, incubated for 20 min, and centrifuged again at 12,000 g (2 min). The final supernatant was recovered as the nuclear protein fraction. All procedure was performed at 4 °C, and the total protein concentration of the samples was determined by the Bradford method (Bio-Rad Laboratories). Samples were stored at -80 °C for further analysis. Equal amounts of nuclear proteins were subjected to immunodetection as described above, using anti-SIRT1 antibody (Cell Signaling Technology, MA, USA mAB#8469).

### Statistical analysis

All data were expressed as mean ± standard error of the mean (SEM). The results of four groups were matched by one-way analysis of variance (ANOVA), with Student-Newman-keuls as a post-test. Differences were considered statistically significant at *P* < 0.05.

## Results

### Resveratrol improved glycemic control

Table 2 shows data related to the metabolic control. As expected, blood glucose and plasma fructosamine concentrations, as well as 24-hour urinary glucose content were significantly higher in diabetic rats (DP vs. ND, *P* < 0.001). In both DI and DIR groups, there was a reduction in blood glucose (*P* < 0.001) to levels similar to those observed in non-diabetic rats. Glycosuria also decreased after insulin treatment (DI vs. DP, *P* < 0.01), and resveratrol induced an additional reduction (DIR vs. DI, *P* < 0.001), although glycosuria still remained higher in comparison to non-diabetic rats. Fructosamine concentration decreased in the DI group, and a further reduction was observed with resveratrol (DIR vs. DI, *P* < 0.001), achieving mean value similar to that observed in non-diabetic rats.

**Table 2:** Characteristics of the non-diabetic (ND), diabetic rats treated with placebo (DP), with insulin (DI) and with insulin plus resveratrol (DIR)

	ND	DP	DI	DIR
Body Weight (g)	416 ± 10.8	294 ± 15.7***	354 ± 7.4**^###^	363 ± 9.5**^###^
Blood glucose (mg/dL)	125.2 ± 2.5	502.1 ± 18.7***	140 ± 27.5^###^	153 ± 31.0^###^
Glycosuria (mg/24 h)	2.7 ± 0.9	234 ± 20.9***	162 ± 23.7***^##^	59.2 ± 5.4*^###§§§^
Fructosamine (μMol/L)	98.7 ± 4.3	205 ± 11.0***	138 ± 4.6**^###^	84.6 ± 11.3^###§§§^

Data are mean ± SEM of nine to ten animals, and were compared by one-way ANOVA, Student Newman-Keuls post test. *P < 0.05, **P < 0.01 and ***P < 0.001 vs ND; ^##^ *P* < 0.01 and ^###^ *P* < 0.001 vs DP; ^§§§^ *P* < 0.001 vs DI

### *Slc2a4* and GLUT4 expression in soleus

**Fig. 1:**
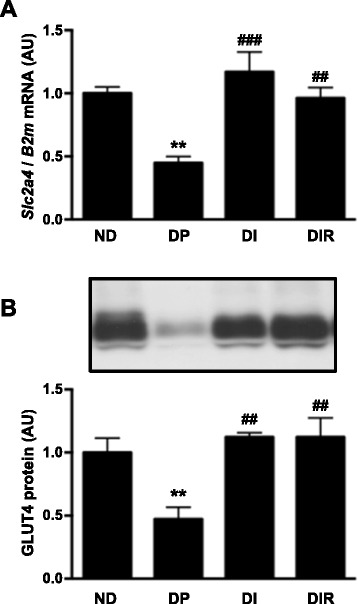
*Slc2a4* mRNA (**a**) and GLUT4 protein (**b**) in soleus skeletal muscle of non-diabetic (ND) and diabetic placebo- (DP), insulin- (DI) and insulin plus resveratrol- (DIR) treated rats. AU, arbitrary units. Data are mean ± SEM of four to six animals. **P < 0.01 vs ND; ^##^ *P* < 0.01 and ^###^ *P* < 0.001 vs DP. One-way analysis of variance (ANOVA), Student-Newman-Keuls post-test

To investigate the skeletal muscle regulation of glucose disposal, and its participation in glycemic regulation, the *Slc2a4* mRNA and its GLUT4 protein were measured in soleus muscle (Fig. 1). Both mRNA, and protein reduced by ~55 % in muscles from diabetic rats (*P* < 0.01 vs. ND). Insulin treatment restored both mRNA and protein expression, and resveratrol did not alter the effect of insulin.

### Resveratrol decreased GLUT2 expression in liver, but not in intestine and kidney

**Fig. 2:**
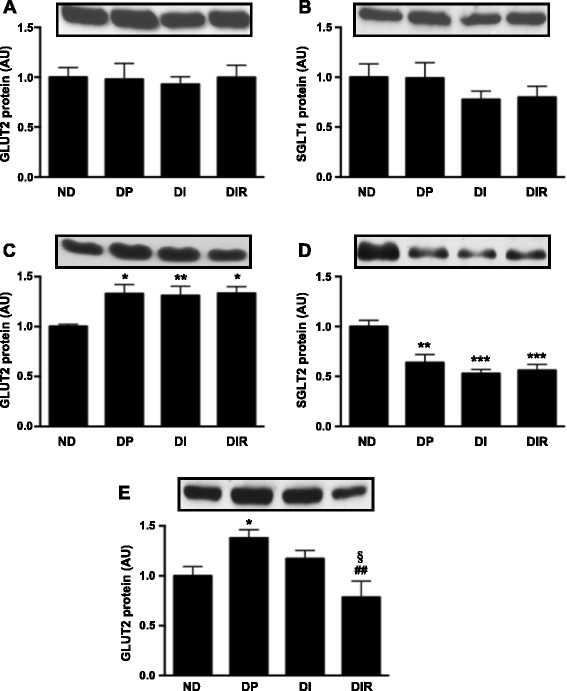
GLUT2 protein in proximal small intestine (**a**), renal proximal tubule (**c**) and in liver (**e**); SGLT1 protein in proximal intestine (**b**) and SGLT2 protein in renal proximal tubule (**d**) of non-diabetic (ND) and diabetic placebo- (DP), insulin- (DI) and insulin plus resveratrol- (DIR) treated rats. AU, arbitrary units Data are mean ± SEM of five to nine animals. *P < 0.05, **P < 0.01 and ***P < 0.001 vs ND; ^##^ *P* < 0.01 vs DP; ^§^ *P* < 0.05 vs DI. One-way analysis of variance (ANOVA), Student-Newman-Keuls post-test

To investigate other territorial fluxes of glucose which could be involved in the resveratrol-induced whole-body improvement of glycemic control, expression of glucose transporters was evaluated in the proximal small intestine (Fig. 2a and b), renal proximal tubule (Fig. 2c and d), and liver (Fig. 2e). The GLUT2 expression did not change in the proximal small intestine in any experimental condition, as well as its correspondent luminal sodium-glucose coupled transporter SGLT1. In renal proximal tubule, DM increased GLUT2 (DP vs. ND, *P* < 0.05), an effect that was not significantly reversed by insulin with or without resveratrol. Differently, the expression of the luminal SGLT2 in proximal tubule was reduced by DM (DP vs. ND, *P* < 0.01), which also was not altered by insulin with and without resveratrol. Finally, in liver, GLUT2 protein increased in response to DM (DP vs. ND, *P* < 0.05), insulin treatment tended to reduced it, but a significant reduction was observed only with the administration of resveratrol to insulin therapy (*P* < 0.01 vs. DP and *P* < 0.05 vs. DI).

### Resveratrol regulates glucose metabolism markers in liver

**Fig. 3:**
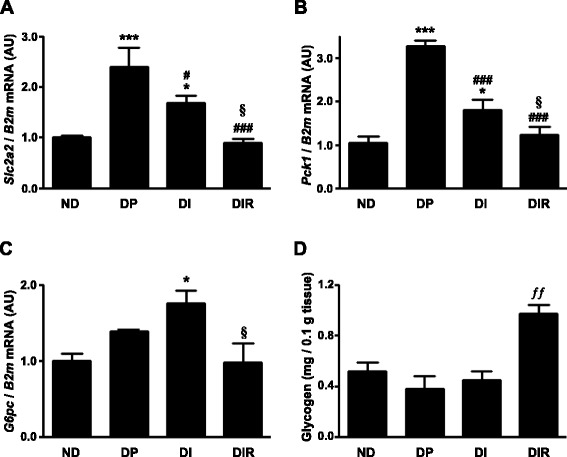
*Slc2a2* (**a**) *Pck1* (**b**) and *G6pc* (**c**) mRNA expression and glycogen content (**d**) in liver of non-diabetic (ND) and diabetic placebo- (DP), insulin- (DI) and insulin plus resveratrol- (DIR) treated rats. AU, arbitrary units. Data are mean ± SEM of five to seven animals. *P < 0.05 and ***P < 0.001 vs ND; ^###^ *P* < 0.001 vs DP; ^§^ *P* < 0.05 vs DI; ^ƒƒ^ *P* < 0.01 vs all groups. One-way analysis of variance (ANOVA), Student-Newman-Keuls post-test

*Slc2a2* mRNA (Fig. 3a) was modulated exactly as the GLUT2 protein in the liver. DM increased *Slc2a2* mRNA, insulin treatment partially reduced it, but only the adjunctive treatment with resveratrol restored it to non-diabetic levels (DIR vs. DP, *P* < 0.001; DIR vs. DI *P* < 0.05I).

The expression of *Pck1* mRNA (Fig. 3b) was similarly regulated. DM increased *Pck1* mRNA (~3.2-fold, *P* < 0.001 vs. ND), insulin treatment partially reduced it (*P* < 0.001 vs. DP), and that was exacerbated with resveratrol (*P* < 0.001 vs. DP; *P* < 0.05 vs. DI), which brought mean expression to the level observed in non-diabetic animals.

*G6pc* mRNA (Fig. 3d) tended to increase in diabetic rats, and insulin therapy made that significant (DI vs. ND, *P* < 0.05). Importantly, the resveratrol treatment reversed the insulin-induced increase in *G6pc* mRNA (DIR vs. DI, *P* < 0.05), restoring its expression to the level observed in non-diabetic rats.

Finally, the hepatic glycogen content (Fig. 3d) was not altered by insulin therapy, but was recovered by the adjunctive treatment with resveratrol (*P* < 0.001 vs. DP; *P* < 0.01 vs. DI), becoming higher than in non-diabetic rats (*P* < 0.01 vs. ND).

### Resveratrol increased nuclear SIRT1 content in liver

**Fig. 4:**
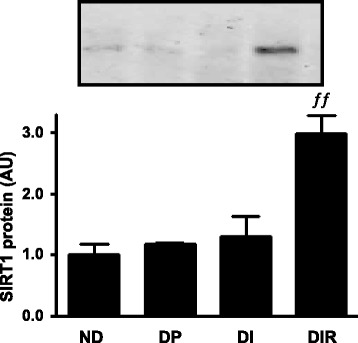
Nuclear SIRT1 protein in liver of non-diabetic (ND) and diabetic placebo- (DP), insulin- (DI) and insulin plus resveratrol- (DIR) treated rats. AU, arbitrary units. Data are mean ± SEM of 3 animals. ^ƒƒ^ *P* < 0.01 vs all groups. One-way analysis of variance (ANOVA), Student-Newman-Keuls post-test

Resveratrol effects have been proposed to be mediated by SIRT1, which, once activated in the nucleus, may impair transcriptional activity. The nuclear content of SIRT1 in the liver was unaltered in diabetic rats insulin-treated or not (Fig. 4). However, the adjunctive treatment with resveratrol induced a 3-fold increase in the nuclear content of SIRT1 (DIR vs. all groups, *P* < 0.01).

## Discussion

The present study was carried out to investigate if resveratrol would improve glycemic control in T1D-like rats under insulin therapy. As expected, insulin treatment did not completely restore glycemic control, because it is difficult to mimic endogenous insulin secretion [5], which is also observed in T1D patients [33]. Resveratrol revealed a powerful adjunctive effect, being able to induce a strong additional reduction in glycosuria and to bring fructosamine concentrations to values similar to those observed in non-diabetic rats. This improvement in glycemic homeostasis seems to be related to increased insulin sensitivity in the liver, with a consequent reduction in glucose efflux.

Streptozotocin (STZ) treatment of rats is known to induce a specific pancreatic beta cell necrosis, leading to an insulinopenia syndrome [34], similar to the immunologic induced beta cell necrosis in T1D human, except by the extension of the beta cell lack, which is more pronounced in humans.

Several reports have proposed that resveratrol improves glycemic control in animals and humans with DM [11], most of them conducted in T2D [11, 15, 17, 35]. Besides improvement in insulin sensitivity, an improvement in insulin secretion has also been reported to participate in resveratrol effects [15, 16]. Thus, potential benefits of resveratrol in T1D should be limited. However, some studies were performed in T1D-like STZ rats not treated with insulin, and a resveratrol-induced effect was clearly detected, such as reduction in blood glucose from 525 to 450 mg/dL [18] and from 469 to 373 mg/dL [19]. These data highlight the insulin sensitizer effect of resveratrol, but its use in T1D without concomitant insulin therapy would be proscribed. Considering that, we evaluated resveratrol as adjunct to insulin therapy in T1D, which has never been investigated.

The present results revealed the impressive effect of resveratrol as adjunct to insulin therapy in T1D-like rats based on fructosamine decay. The serum fructosamine includes all glycated plasmatic proteins produced over the last 2-3 weeks, reflecting recent changes in glycemic control. Since peripheral insulin concentration is unchangeable in this T1D model, the glucose-lowering effect must have been achieved due to changes in one or more peripheral flux of glucose, which invariably is accompanied by changes in the glucose transporters expression.

Participation of improved skeletal muscle glucose uptake by resveratrol has already been proposed. Piceatannol, a metabolite of resveratrol, was shown to increase AMPK phosphorylation, GLUT4 translocation, and glucose uptake in L6 myocytes [12]. Besides, in muscle from 2-week STZ-diabetic rats not receiving insulin, 7-day resveratrol treatment increased soleus GLUT4 content to a value similar to that observed in insulin-treated animals [14]. We observed a decreased *Slc2a4*/GLUT4 expression in diabetic rats, and that was completely recovered by insulin treatment, as reported elsewhere [5, 36]. Resveratrol administration did not modify muscle *Slc2a4*/GLUT4 expression in comparison to insulin alone, showing that, as adjunctive therapy in T1D, this compound does not improve glucose clearance by skeletal muscle.

SGLT1- and GLUT2-related increase in intestinal glucose absorption has been proposed in T2D patients [37] and 2-week STZ-diabetic rats [38]. However, modulation in SGLT1 and/or GLUT2 in T1D-like long-term models has never been clearly reported. In the present study, we did not observe alterations in either SGLT1 or GLUT2 proteins in diabetic rats, regardless of the treatment employed, suggesting that modifications in the intestinal glucose absorption rate do not contribute to the observed changes in glycemic homeostasis induced by resveratrol. We did not find previous studies which evaluated the effects of resveratrol in intestinal SGLT1 and GLUT2 expression.

It has been extensively reported that inhibition of renal glucose reabsorption by SGLT2 inhibitors contributes to blood glucose reduction, and that is a recent therapeutic approach to DM treatment [27]. STZ-diabetic rats revealed increased *Slc5a2* mRNA expression [39]; however, SGLT2 protein expression was not investigated. On the other hand, in mice, DM-induced regulation of SGLT2 is controversial, being reduced in STZ-diabetic mice, but increased in *db/db* and Akita mice [40], pointing out to the complexity of this regulation. In the present study, 7 weeks of DM duration in STZ-diabetic rats decreased SGLT2, and neither insulin nor insulin plus resveratrol altered this pattern of expression. Differently, GLUT2 has been clearly described to increase after 4 weeks of DM [28, 31], as observed here; and insulin reversed this effect. Overall, since resveratrol altered neither SGLT2 nor GLUT2 expression, there is no data to support a resveratrol-induced reduction in renal glucose reabsorption, which could contribute to improving glycemic control.

Relevant results were obtained in the liver. GLUT2 protein increased in diabetic rats and was partially reduced by insulin therapy, in agreement with previous work [31]. Remarkably, the adjunctive treatment with resveratrol promoted a further decrease in GLUT2 content, restoring the protein levels to those of non-diabetic rats. Besides, parallel regulations were detected in *Slc2a2* mRNA expression, indicating a transcriptional modulation. Since a reduction in GLUT2 expression has been related to reduced hepatic glucose efflux in several conditions, such as the Fanconi-Bickel syndrome [41] and the Foxa3^-/-^ mice [42], this result points out the hepatic territory as responsible for the beneficial effects of resveratrol.

To deepen the investigation of liver participation, some markers of glucose metabolism, which have been related to impaired glycemic homeostasis in DM [43], were analyzed. The expression of the pivotal gluconeogenic enzyme *Pck1*, which increased in DM, was only restored to non-diabetic levels by treatment with insulin plus resveratrol. *G6pc* expression tended to increase in DM, and insulin therapy did not reverse this effect, as it was expected, considering the repressor effect of insulin on *G6pc* gene [44]. However, the resveratrol treatment restored the *G6pc* expression to the non-diabetic levels. These findings reveal the resveratrol-induced increase in the local insulin sensitivity, and anticipate a reduction in hepatic glucose production and efflux [45, 46]. Indeed, in the liver of diabetic rats not treated with insulin, resveratrol was reported to increase the insulin signaling pathway activity [47], and restored PEPCK expression [14] and G6Pase activity [48]. However, resveratrol effect upon hepatic glucose metabolism, as adjunctive therapy to insulin in T1D had never been previously investigated.

Increased deacetylase activity of SIRT1 was extensively proposed as the major mechanism by which resveratrol induces beneficial effects in several tissues [11]. However, in the liver, this effect was far from being demonstrated. Indeed, in the liver of STZ-diabetic rats not receiving insulin, resveratrol increased *Sirt1* mRNA [18], but had no significant effect on the cytosolic protein [49]; besides, in the liver of insulin resistant KKAy mice, an increase in SIRT1 protein, supposedly measured in a homogenate, was reported to be increased in only one mouse [50]. We are showing for the first time a resveratrol-induced robust increase in nuclear SIRT1 protein, which might be involved in the regulation of the hepatic glucose metabolism-related genes investigated.

Interestingly, despite proposals that resveratrol-induced improvement in glycemic homeostasis could involve suppression of gluconeogenic enzymes expression [11, 48], and that this effect could be mediated by increased SIRT1 activity [11, 18, 49, 50], previous studies suggested the opposite. Activation of SIRT1 in isolated hepatocytes was reported to increase [51], whereas SIRT1 knockdown in mice was reported to decrease [52] the expression of key gluconeogenic enzymes. Thus, it remains to be further investigated whether there is a cause-effect relationship between increased nuclear SIRT1 and decreased *Pck1*/*G6pc* expression in the liver of T1D-like rats receiving resveratrol plus insulin.

SIRT1-mediated decrease of insulin resistance has been proposed, mainly based on resveratrol treatment [11, 50], although the participation of SIRT1 as a nuclear deacetylase is far from being clearly demonstrated in these studies. In the present study, the resveratrol-induced amelioration of glycemic control depicts improvement of insulin sensitivity, since the T1D animals were submitted to the same insulin regimen. Besides, the improvement in insulin sensitivity was observed in liver, where regulation of genes related to glucose metabolism was observed, together with increased nuclear content of SIRT1. Thus, for the first time, the insulin sensitizer effect of SIRT1 deacetylase can be rationally proposed.

## Conclusion

In conclusion, resveratrol was able to improve glycemic control in insulin-treated T1D-like rats. This effect seems not to involve changes in glucose fluxes in the small intestine, renal proximal tubule, and soleus skeletal muscle; but to be related to several changes in the liver, where downregulation of *Slc2a2*/GLUT2, *Pck1* and *G6pc* expression was observed, favoring reduction of glucose production and efflux. Besides, resveratrol increased SIRT1 nuclear protein content in liver, which may be related to the observed gene expression regulations.

## Abbreviations

AMPK, AMP-activated protein kinase; ANOVA, analysis of variance; AU, arbitrary units; *B2m*, beta-2-microglobulin gene; DI, insulin-treated diabetic rat; DIR, insulin + resveratrol-treated diabetic rat; DM, diabetes mellitus; DP, placebo-treated diabetic rats; G6Pase, glucose-6-phosphatase protein; *G6pc*, glucose-6-phosphate catalytic subunit gene; GLUT2, glucose transporter 2 protein; GLUT4, glucose transporter 4; ND, non-diabetic rat; *Pck1*, phosphoenolpyruvate carboxykinase gene; PEPCK, phosphoenolpyruvate carboxykinase protein; qPCR, real-time polymerase chain reaction; RT, reverse transcriptase reaction; SEM, standard error of the mean; SGLT1, sodium-glucose cotransporter 1; SGLT2, sodium-glucose cotransporter 2; SIRT1, sirtuin 1; *Slc2a2*, solute carrier family 2 member 2 gene; *Slc2a4*, solute carrier family 2 member 4 gene; STZ, streptozotocin; T1D, type 1 diabetes mellitus; T2D, type 2 diabetes mellitus
